# Hearing handicap and frailty in community-dwelling older adults living

**DOI:** 10.1590/2317-1782/20212021080

**Published:** 2022-04-08

**Authors:** Ruana Danieli da Silva Campos, Marisa Silvana Zazzetta, Fabiana de Souza Orlandi, Sofia Cristina Iost Pavarini, Márcia Regina Cominetti, Ariene Angelini dos Santos-Orlandi, Isabela Thaís Machado de Jesus, Grace Angélica de Oliveira Gomes, Aline Cristina Martins Gratão, Letícia Pimenta Costa-Guarisco

**Affiliations:** 1 Programa de Pós-graduação em Gerontologia, Universidade Federal de São Carlos – UFSCar - São Carlos (SP), Brasil.; 2 Departamento de Gerontologia, Universidade Federal de São Carlos – UFSCar - São Carlos (SP), Brasil.; 3 Departamento de Enfermagem, Universidade Federal de São Carlos – UFSCar - São Carlos (SP), Brasil.; 4 Programa de Pós-graduação em Enfermagem, Universidade Federal de São Carlos – UFSCar - São Carlos (SP), Brasil.

**Keywords:** Aged, Hearing Loss, Presbycusis, Frailty, Frail Elderly

## Abstract

**Purpose:**

To verify the relationship between hearing handicap and frailty in community-dwelling older adults.

**Methods:**

A cross-sectional study was carried out with 238 older adults (aged ≥ 60 years) in 2018. The Hearing Handicap Inventory for the Elderly – Screening version – HHIE-S was applied to assess the hearing handicap. To assess frailty, the Frailty Phenotype proposed for Fried and co-workers was adopted, objectively evaluating 5 criteria: unintentional weight loss, reported fatigue, reduced grip strength, reduced walking speed and low physical activity. It was investigated whether the hearing handicap were related with frailty using Kruskal-Wallis and Spearman test.

**Results:**

Worse perception of the hearing handicap was found in pre-frail and frail individuals, compared to non-frail individuals. In addition, hearing handicap showed a positive and statistically significant correlation with frailty.

**Conclusion:**

Hearing handicap is related to frailty in community-dwelling older adults.

## INTRODUCTION

Hearing loss associated with aging is a sensorineural impairment of the hearing organ and hearing pathways. Despite being a physiological change resulting from cochlear aging, many factors favor its occurrence, such as genetic, environmental (exposition to intense noise and ototoxic substances), or individual (ear infection pre-pathways and systemic diseases or comorbidities) components, which explains its prevalence in the population of older adults^(1)^.

According to the Global Burden of Disease Study of 2021, hearing loss affects around 26% of the world population, thus characterizing as a high-prevalence disease and the third main cause of coping with deficiency^(2)^. Regarding the older adults population, international studies have pointed to a prevalence of self-reported hearing complaint in around 20 to 30% of older individuals^(3,4)^, a scenario that is not different in Brazil^(5)^. However, studies based on audiometric measures have found higher prevalence, above 80%, in the older population^(3)^. Although the symptoms are not noticed at first, hearing loss has a slow, gradual, and irreversible progression, leading to the impairment of hearing perception and speech understanding along the years, severely compromising the verbal communication process in the older person, and affecting their life quality^(1)^.

Nonetheless, in addition to changes in the communication process, other problems also result from hearing impairment in older individuals, such as social isolation, feelings of devaluation, decreased self-esteem, depression, and difficulties in family relations and in using means of communication^(1)^. Therefore, it is crucial to detect not only hearing impairment, but hearing handicap as well.

According to the WHO, a deficiency is an abnormality in the organs, systems, or body structures, while an impairment is the implication of a deficiency from the functional point of view. Handicap, in turn, reflects the adaptation of an individual to their deficiency or impairment. In light of this, the International Functional Classification (IFC) describes both the nature and severity of functionality limitations^(6)^. According to the IFC, an hearing deficiency leads to an hearing impairment and consequent handicap, which may either limit or restrict the individual’s participation in daily life activities^(7)^. Thus, it can be said that an hearing loss may prevent or limit the older person to fully perform their activities, therefore being regarded as one of the most frustrating sensory deprivation and disabling chronic disorders that affect both the functionality and life quality of older adults^(8)^.

For such reasons, as the population ages, associated hearing losses become a common concern to public health, as well as frailty. Many instruments have been elaborated to assess the restriction of participation, including the widely used HHIE-S, aimed at the older adults population. The instrument was proposed to assess handicap in the older adults population, that is, the individual’s own perception of their hearing limitation and how it affects their lifestyle, family relations, and social and emotional situation. These questionnaires are also important to assess the promotion of hearing health services for allowing to monitor the influence of impairments and handicaps on the life quality of older individuals^(9)^.

Frailty, in turn, is a clinical syndrome resulting from declines in the physiological reserve associated to the impairment, in addition to reduced neurological control, mechanical performance, and energy metabolism^(10)^. Frailty implies loss of homeostatic capacity to resist stressors, causing vulnerability in the systems^(11)^. Nonetheless, the loss of reserve in multiple systems may lead to a decreased capacity of the organism to tolerate stressors, thus increasing the risk of negative outcomes associated with frailty, such as hospitalization, early institutionalization, falls, functional loss, and death^(11)^. Fried et al.^(11)^ proposed five criteria to detect frailty that indicate energy impairment, like muscle weakness, fatigue or exhaustion, slow walking speed, reduced physical activity, and unintentional weight loss.

Recent studies have suggested that hearing loss is related to frailty and its implications^(4,12,13)^. Early results, published in 2014^(12)^, showed an association between self-reported hearing loss and frailty in older women, which did not apply to men. Hearing loss has been associated with risk of developing frailty and falls along the years^(13)^, and the risk of frailty progression was corroborated in subsequent studies^(4,14)^.

Although it is a topic of great interest in the field of aging, it is noteworthy that studies addressing the relationship between hearing loss and frailty are still recent and scarce in the literature. Background studies used a single question regarding hearing self-perception^(4,12)^ or results from audiometric assessments to investigate hearing issues^(13)^. Nonetheless, there are no studies that applied the HHIE-s^(15)^, an instrument validated and consolidated in the international literature for measuring handicap or restricted hearing participation, as well as social and emotional damages resulting from the hearing loss. Therefore, our goal is to verify whether there is a relationship between hearing handicap and the presence of frailty in older adults of the community.

## METHODS

### Ethical issues

This study used data derived from the project “Monitoring tool for frailty levels in older individuals served by the basic health care: assessment of effectiveness and efficiency”, conducted by the research group Aging Management – GEnv. The project was authorized by the Municipal Secretariat of Health and approved by the Research Ethics Committee of the Federal University of São Carlos (CAAE:861067418.4.0000.5504 and Decision nº 3.101.282), in accordance with the ethical expectations in the Resolution 466/2012 regulated by the National Health Council.

### Design, study site, and period

This is a cross-sectional, observational study of quantitative approach carried out in the context of a large community of high social vulnerability, “*Cidade Aracy*”, in the city de São Carlos-SP. The research took place between 2017 and 2018 through home assessments of older adults registered in five units of the Family Health Units (USFs – abbreviation in Portuguese) supported by the regional health administrations (ARES – abbreviation in Portuguese).

### Sample, inclusion and exclusion criteria

The following inclusion criteria were applied: individual aged 60 years or more, registered in the USFs of the ARES “*Cidade Aracy*” served by the family health center, able to understand and communicate verbally, and acceptance of participating in the study and signature of the Informed Consent Form. The following exclusion criteria were applied: conditions that would prevent the tests from being performed, such as severe motor deficits, lack of communication or terminally ill status. The final sample included 238 older adults who met all eligibility criteria.

### Study protocol

Our research protocol is constituted of sociodemographic characterization data, frailty test, and hearing handicap inventory. The sociodemographic and health data were collected by means of a questionnaire elaborated by the researchers encompassing information on sex, age, race, marital status, education, income, and comorbidities.

For frailty data, we used the phenotype proposed by Fried et al.^(11)^, which assesses frailty according to the following five components: 1) Unintentional weight loss – assessed based on the following question: “Over the last twelve months, do you consider having lost weight without undergoing any diet?” In case of affirmative answer, if the weight loss is equal or higher than 4.5 kg or 5% of body weight over a year, the criterion was met. (2) Fatigue – assessed through self-reported based on the following two questions: “Have you felt the need to make any effort to cope with your daily tasks?” and “Have you felt unable to perform your tasks?”. If the older adults individual answered “always” or “mostly” for any of the two questions, the criterion was met. (3) Low strength of palm grip – measured by instructing the older adults individual to squeeze the device as strong as possible. Before that, the individual would receive the command “attention, get ready, now!” and was stimulated during the test by “go, stronger, go!”. For this criterion, the result was reached in the arithmetic mean of three measures and adjusted according to the Body Mass Index and sex, considering the weakest 20%in the sampling distribution. (4) Low level of caloric expenditure – assessed through self-report based on the following question “Do you consider doing fewer physical activities than twelve months ago?” In case of affirmative answer, the criterion was met. (5) gait slowness – assessed by the average time spent to walk 4.6 meters of distance, with adjustment according to sex and height. Three measures of walking speed were performed using the arithmetic mean as response considering the slowest 20% in the sampling distribution. According to the score achieved in the five criteria, the older adults individual would be classified according to frailty^(11)^ as: non-frail (no score in none of the five criteria), pre-frail (score in one or two criteria), or frail (score in three or more criteria).

To assess the hearing handicap, we applied the Hearing Handicap Inventory for the Older adults – Screening version (HHIE-S)^(15)^ questionnaire, an instrument translated and validated to the Brazilian context since 1997^(16)^. It is an instrument of hearing screening for older adults individuals that assesses the perception of hearing handicap (impairment or restriction to hearing participation), as well as social and emotional impact resulting from the perceived hearing loss. Composed of ten questions and divided between social and emotional scales, each item has the following response options: yes (4 points), sometimes (2 points), or no (0 points). Total scoring varies from 0 to 40 points, classified into three categories: 0-8 points – no perception of hearing handicap, 10-22 points – slight or moderate perception, and 24-40 points – significant perception of hearing handicap.

### Result analysis and statistics

The protocols were checked before typing the data of the main study. Data were then typed in two different spreadsheets by different researchers blindly on the MS Excel XP software and further imported and analyzed using the IBM SPSS Statistics 20 statistical package.

For the sampling profile description, we performed descriptive statistics with measures of position and dispersion (mean, standard deviation, minimum and maximum values, median) for the continuous variables. Tables of absolute and relative frequencies were elaborated for the category variables. Data normality was assessed through a Komogorov Smirnov test, followed by Kruskal-Wallis non-parametric tests to assess the relationship between hearing handicap and frailty using the mean values of the HHIE-S to compare the groups according to frailty based on Spearman Correlation to assess the correlation between hearing handicap and frailty (continuous variables) by adopting the following scale to interpretate statistically significant values: weak <0.29, moderate de 0.3 a 0.59, strong de 0.6 a 0.9, and optimum 1.0^(17)^. All analyses considered p≤ 0.05 as statistical significance.

## RESULTS

The sample included 238 older adults aged between 60 and 100 years, average of 71.9 years, out of which 85.7% were aged below 80 years. It is a low-income population with poor educational level, residents of a community regarded as to be highly socially vulnerable. The conditions of frailty and pre-frailty appeared in most of the sample, while 26.9% of the population studied reported some perception of hearing handicap (Table 1).

**Table 1:** Sociodemographic and health characterization. São Carlos, São Paulo, Brazil, 2018 (n=238)

Variable	Category	n	%	Mean	SD	Median	[Min-Max]
Sex	Female	99	41.6				
	Male	139	58.4				
Age (years)				71.1	7.3	71	[60-100]
Age group	60-610 years	104	43.7				
	71-710 years	100	42				
	80+ years	34	14.3				
Race/ethnicity	White	117	410.2				
	Non-white	121	50.8				
Education (years)				2.6	2.8	2	[0-17]
No. of people living in the residence	1-3 people	191	80.3				
	4-5 people	40	16.8				
	6-8 people	7	2.1				
Family outcome	R$800 to R$ 1200	41	17.2				
	R$1201 to R$ 2100	66	27.7				
	R$2101 or more	67	28.2				
	No answer	64	26.1				
			
Comorbidities	Hypertension	169	71				
	Diabetes *Melittus*	166	29.4				
	Osteoporosis	38	16				
	Depression	31	13.1				
	Stroke	23	10.5				
	Cancer	12	5.5				
Hearing Handicap (HHIE-S)*	No damage	174	73.1				
	Damage level/ moderate	49	20.6				
	Damage significant	15	6.3				
Frailty	Non-frail	55	23.1				
	Pre-frail	134	56.3				
	Frail	49	20.6				

* Hearing Handicap Inventory for the Older adults – Screening version^(15)^

Table 2 provides the mean score of the HHIE-S test per group according to the frailty criterion. We found significant differences regarding the mean score of the HHIE between non-frail and frail older adults, as well as between non-frail and pre-frail older adults individuals.

**Table 2:** Comparison between levels of hearing handicap and frailty. São Carlos, São Paulo, Brazil, 2018 (n=238)

	Non-Frail	Pre-Frail	Frail
N	55	134	49
Mean HHIE-S*	3.7	6.8	8.9
SD	7.8	9.5	10.8
NF/P^Fa^ p-value	0.044**		
NF/Fa p-value	0.015**		
PF/Fa p- value	0.995		

a Kruskal Wallis Test

* Hearing Handicap Inventory for the Older adults - Screening version^(15)^;

** Statistically significant

We applied the Spearman correlation test to verify whether there was statistically significant correlation between hearing handicap and frailty. The outcome showed a positive correlation between the variables both for the social and emotional scales since the increase in the HHIE-S test score was followed by a higher frailty score (r=0.218; p=0.001) (Table 3).

**Table 3:** Correlation between hearing and frailty damages. São Carlos, São Paulo, Brazil, 2018 (n=238)

Damage hearing (HHIE-S)*	Frailty	
	*r*	*p*
	0.218	0.001**
Social Scale	0.228	0.001**
Emotional Scale	0.2	0.002**

Spearman Correlation

* Hearing Handicap Inventory for the Older adults – Screening version^(15)^;

** Statistically significant

## DISCUSSION

This research aims to investigate the relationship between hearing handicap and frailty in 238 older adults individuals of the community. The results demonstrate that frail and pre-frail older adults reported restricted participation in daily life activities according to the questionnaire HHIE-S. Such result is ratified by the positive correlation found between frailty score, according to Fried Phenotype, and the HHIE-S score.

The HHIE-S instrument used herein aims to quantify the social and emotional impacts of hearing loss and is useful for hearing screening and measurement of treatment responses and follow-up, both in the clinical scope and research^(18)^. Like the presence of self-reported hearing complaint, hearing handicap research is a subjective approach to hearing assessment commonly used in international^(4)^ and national^(5)^ population studies with household collections aimed at investigating restrictions due to hearing problems. The questions have been validated in previous studies whose accuracy has been tested in relation to other objective assessment approaches^(16,18)^.

With the publication of the IFC in 2001 and its larger use along these past two decades, hearing loss now has a biopsychosocial approach focused on health and welfare promotion, with the impairment developed within the individual-environment interaction. Tools proposing the assessment of hearing self-perception in daily life activities gained higher relevance for allowing to assess the restriction to hearing participation^(9,19)^. Despite their unique relevance, Tonal Threshold Audiometry and other hearing sensitivity measures are not sufficient to measure hearing handicap since they do not address how the individual adapts to their deficiency or the impact on life quality. Thus, investigating hearing handicap and its relationship with frailty in older adults individuals implies more than a mere hearing loss detection but regards the individual as a biopsychosocial being within a specific environmental context, with skills to adapt to their deficiency/impairment, thus justifying the relationship found between restriction of hearing participation and frailty in older adults.

Frailty was investigated through the instrument proposed by Fried et al.^(11)^ pointing to a high number of pre-frail and frail older adults, numbering 76.10% of the sample – a similar prevalence to other studies in both the national and international literature based on the same frailty assessment instrument in the community^(13,20)^.

By investigating the relationship between the HHIE-S and frailty, we found that pre-frail and frail individuals have higher restriction of participation in daily life activities than non-frail individuals (p=0.044 and 0.015, respectively). However, the pre-frail and frail groups did not differ when compared to the HHIE-S mean. It is noteworthy that longitudinal studies have pointed to higher incidence risk of hearing loss and frailty progression^(4,12,14)^.

Accordingly, the results show a statistically significant positive correlation between the HHIE-S score and frailty score (r=0.218 and p=0.001) both in the social and emotional scales of the HHIE-S. Although the correlations had weak magnitude, these findings suggest that the greater the restriction in participation in daily life activities the higher the score for frailty, thus showing the strength of this similar correlation in social and emotional scales.

Our study shows to be very up to date since the literature has very few publications on the topic of hearing loss and frailty and our results are aligned with background findings^(4,12,13,21)^, thus demonstrating the relevance of the research question. We found no national studies or in Portuguese addressing the topic. The oldest study detected in the literature directly addressing the topic of frailty and hearing loss was published in 2014^(12)^ followed by a publication in 2015^(13)^ and another in 2017^(4)^. All these studies, despite based on different methodologies, found an association between hearing loss and frailty. However, the authors highlight the adverse health conditions involved, such as diabetes, cardiovascular diseases, or history of falls as factors associated with frailty^(13)^ and/or hearing losses^(4,22)^. Potential causes of such an association include a shared neuropathological etiology (like microvascular disease and inflammation) contributing both to hearing deficiency and frailty^(12)^.

Hearing loss has also been associated with higher death risk by cardiovascular diseases, in addition to an even higher death risk by all causes when associated with other sensorial deficiencies, such as visual, possibly because it is common for older individuals to have multiple sensorial deficiencies, which may cause adverse health conditions and increases frailty risk and consequently death^(12,22,23)^.

The association between hearing loss and death was also investigated^(23)^, and the conclusion is that hearing loss in older adults may be associated with an increase by 20% in the death risk, which can be explained by shared pathological processes like mitochondrial dysfunction, generalized inflammation, or microvascular disease potentially evolving to hearing loss and death risk.

Other sociodemographic variables such as age, sex, income, education, and social support were also considered in earlier studies on hearing loss and frailty. A cross-sectional study with 2,1010 older adults individuals over 70 years considered a stratification by sex found that the hearing was significantly associated with frailty in women, but not in men^(12)^.

A cohort study on the topic was published in 2017^(4)^ based on a self-assessment question for detecting hearing loss, and Fried phenotype for frailty, therefore a pioneer study. The research monitored 2,836 older adults individuals whose hearing was self-assessed by means of a single question to be answered as excellent, very good, good, reasonable, or poor. The responses were arranged in order to constitute two analysis groups: good hearing (excellent, very good, good response) and poor hearing (reasonable or poor response). The research analyzed baseline data cross-sectionally and reassessed the older adults individuals after four years. To establish the association between hearing loss and frailty, the researchers considered adjustments of sex, age, income, education, diseases cardiovascular, cognition, depression, and socialization and found an association of hearing loss with frailty and pre-frailty in the base study (cross-sectional cut). Such an association was confirmed by the longitudinal analysis showing a relationship between hearing loss in pre-frail older adults individuals with higher risk of frailty progression, regardless of covariables. For the authors, hearing loss in older adults individuals may be a frailty risk, suggesting that hearing deficiency accelerates frailty progression^(4)^.

The communication limitations imposed by hearing loss cause social and family isolation due to a restriction of hearing participation and in daily life activities, like going for groceries, to the church, meetings etc. Such a scenario not only generates impacts on social activities but also reduces the levels of physical activity and life quality^(24)^. Thus, it is crucial to consider the necessary interventions, such adapting the Individual Sound Amplification Device (ISAD) for hearing recovery seeking to improve self-perception of handicap and life quality of older adults^(25)^.

Finally, we found that the most prevalent comorbidities were hypertension (71%) and diabetes mellitus (29.4%). Sociodemographic features as age, sex, education, income, and ethnicity were also addressed in this study, although not considered for analyzing the factors associated with hearing loss or frailty, thus implying a research limitation.

Therefore, although previous studies have shown that the relationship between hearing loss and frailty persists even after the adjustments of the associated factors, our results do not allow us to state that the relations found between the two variables are not masked by sociodemographic factors or health conditions that interfere both with hearing and frailty in older adults individuals. Accordingly, the cross-sectional nature of our study could not indicate any causality relationship between the two variables, as cohort studies do^(4,12)^.

Nevertheless, it is suggested that such a relation may result from the negative outcomes of hearing loss, like restricted participation in daily life activities, communication difficulty, and consequent social isolation, leading to the onset and progression of frailty^(13)^. Thus, our research proves relevant for corroborating the relationship between hearing and frailty losses – a latest, very little explored topic –revealing the relevance of hearing monitoring in older adults individuals. The restricted participation in daily life activities associated with hearing loss can also represent an important assessment component when identifying individuals with frailty risk^(4)^.

These findings provide many research routes for exploration. We suggest analyzing how the five components included in the frailty phenotype according to Fried are individually related to hearing.

The results analyzed herein are based on hearing self-assessment, more specifically regarding the detection of hearing handicap, that is, restricted participation in daily life activities and related social and emotional impacts resulting from the perceived hearing deprivation. However, the Tonal Threshold Audiometry is considered a gold standard hearing assessment that detects hearing loss and measures its degree. Prior studies point to a lower prevalence of self-declared hearing losses in population studies than hearing losses analyzed through audiometric assessments^(18)^.

Despite the promising results, we do not really know the hearing features that are in fact related to frailty regarding speech degree and understanding. We also did not know how hearing recovery, more specifically the use of hearing aids, could interfere with this relationship. Thus, further research should explore the relationship between frailty and different hearing configurations, as well as how hearing rehabilitation could influence the frailty condition. Prospective studies could deepen the research on the topic by targeting control strategies for frailty progression.

## CONCLUSION

Our results demonstrate the presence of a relationship between subjective perception of hearing handicap and frailty in older population of the community. The relevance of this study is its innovation in correlating hearing loss, more specifically the restricted participation in daily activities, with frailty in vulnerable older adults registered in the basic care service. Another leverage of this research is enabling the health service to use our findings to develop a health monitoring of the older adult individuals aimed at integral care assurance.
