# Supplementary material for: Hearing handicap and frailty in community-dwelling older adults living
Source: Codas. 2022 Apr 8;34(4):e20210080. doi: 10.1590/2317-1782/20212021080 (PMC9886116; doi:10.1590/2317-1782/20212021080)
Supplement: Translation

# Translation: *Handicap* auditivo e fragilidade em idosos da comunidade

**Authors:** Ruana Danieli da Silva Campos, Marisa Silvana Zazzetta, Fabiana de Souza Orlandi, Sofia Cristina Iost Pavarini, Márcia Regina Cominetti, Ariene Angelini dos Santos-Orlandi, Isabela Thaís Machado de Jesus, Grace Angélica de Oliveira Gomes, Aline Cristina Martins Gratão, Letícia Pimenta Costa-Guarisco

**Affiliations:** 1 Programa de Pós-graduação em Gerontologia, Universidade Federal de São Carlos – UFSCar - São Carlos (SP), Brasil.; 2 Departamento de Gerontologia, Universidade Federal de São Carlos – UFSCar - São Carlos (SP), Brasil.; 3 Departamento de Enfermagem, Universidade Federal de São Carlos – UFSCar - São Carlos (SP), Brasil.; 4 Programa de Pós-graduação em Enfermagem, Universidade Federal de São Carlos – UFSCar - São Carlos (SP), Brasil.

**Keywords:** Idoso, Perda Auditiva, Presbiacusia, Fragilidade, Idoso Fragilizado

## Abstract

**Objetivo:**

Verificar a relação entre o *handicap* auditivo e fragilidade em idosos residentes da comunidade.

**Método:**

Estudo transversal realizado com 238 idosos (idade ≥ 60 anos), no ano de 2018. O questionário *Hearing Handicap Inventory for the Elderly - Screening version* - HHIE-S, foi aplicado para quantificar o *handicap* auditivo. A fragilidade foi avaliada segundo o Fenótipo de Fragilidade proposto por Fried e colaboradores, utilizando os 5 critérios: perda de peso não intencional, fadiga relatada, redução da força de preensão, redução da velocidade de caminhada e baixa atividade física. A relação entre o *handicap* auditivo e a fragilidade foi realizada por meio dos Testes Kruskal-Wallis e Spermann.

**Resultados:**

Maior percepção do *handicap* auditivo foi verificado nos indivíduos pré-frágeis e frágeis, comparados aos não frágeis. O *handicap* auditivo apresentou correlação positiva e estatisticamente significante com maiores níveis de fragilidade.

**Conclusão:**

O *handicap* auditivo está relacionado a fragilidade em idosos da comunidade.

## INTRODUÇÃO

A perda auditiva relacionada ao envelhecimento caracteriza-se pelo comprometimento neurossensorial do órgão da audição e vias auditivas. Apesar de ser uma alteração fisiológica em decorrência do envelhecimento coclear, vários fatores contribuem para sua ocorrência como fatores genéticos, ambientais (exposição a ruídos intensos e a substâncias ototóxicas) e individuais (infecções prévias de ouvido e doenças sistêmicas ou comorbidades), o que torna a perda auditiva prevalente na população idosa^(1)^.

De acordo com o Global Burden of Disease Study, de 2021, a perda auditiva atinge cerca de 26% da população mundial, encontrando-se entre as doenças com maior prevalência, sendo a terceira principal causa de anos convividos com deficiência^(2)^. Na população idosa, estudos internacionais apontam prevalência da queixa auditiva autorrelatada em cerca de 20 a 30% dos idosos^(3,4)^ e no Brasil não é diferente^(5)^. No entanto, estudos que utilizaram medidas audiométricas encontraram prevalência maior, acima de 80% em idosos mais velhos^(3)^. Apesar dos sintomas não serem percebidos no início, a perda auditiva possui progressão lenta, gradual e irreversível, levando à incapacidade de percepção auditiva e também da compreensão da fala ao longo dos anos, o que compromete gravemente o processo de comunicação verbal do idoso, afetando sua qualidade de vida^(1)^.

Porém, além das alterações no processo comunicativo, existem outros problemas decorrentes da incapacidade auditiva em idosos, tais como isolamento social, sentimentos de desvalorização, diminuição da autoestima, depressão e ainda, dificuldades de relacionamento familiar e de utilização dos meios de comunicação^(1)^. Por este motivo, é fundamental verificar não somente a incapacidade auditiva, mas também a desvantagem auditiva (*handicap)*.

Segundo a OMS, a deficiência refere-se à anormalidade nos órgãos, sistemas e estruturas do corpo, enquanto a incapacidade refere-se à consequência da deficiência do ponto de vista funcional. Já a desvantagem reflete a adaptação do indivíduo frente à deficiência e à incapacidade. A partir deste conceito, a Classificação Internacional de Funcionalidade (CIF) descreve a natureza e a gravidade das limitações de funcionalidade^(6)^. À luz da CIF, a deficiência auditiva leva à incapacidade auditiva e consequente desvantagem, o que pode limitar ou restringir a participação do indivíduo em atividades de vida diária^(7)^. Assim, pode-se dizer que a deficiência auditiva pode impedir ou limitar o idoso a desempenhar plenamente suas atividades, sendo considerada, uma das mais frustrantes privações sensoriais e um dos mais incapacitantes distúrbios crônicos que afetam a funcionalidade e a qualidade de vida dos idosos^(8)^.

Por esses motivos, à medida que a população envelhece, as perdas auditivas advindas do envelhecimento tornam-se uma preocupação comum à saúde pública, assim como a fragilidade.

Vários instrumentos já foram desenvolvidos para avaliar a restrição da participação, sendo um dos mais utilizados o HHIE-S, desenvolvido para a população idosa. Este instrumento foi proposto para avaliar o *handicap* da população idosa, ou seja, a percepção do próprio indivíduo a respeito de sua limitação auditiva, e como esta afeta seu estilo de vida, relação familiar, sua situação social e emocional. Esses questionários também são importantes na avaliação dos serviços de promoção da saúde auditiva, pois, por meio de sua aplicação, é possível monitorar como as incapacidades e desvantagens afetam a qualidade de vida dos idosos^(9)^.

A fragilidade, por sua vez, é uma síndrome clínica decorrente de declínios da reserva fisiológica associada à incapacidade e redução do controle neurológico, desempenho mecânico e metabolismo energético^(10)^. A fragilidade envolve perda de capacidade homeostática para a resistência de estressores, resultando em vulnerabilidade dos sistemas^(11)^. Contudo, a perda da reserva em múltiplos sistemas pode levar a um declínio na capacidade do organismo em tolerar estressores aumentando o risco de desfechos negativos associados a fragilidade, tais como, hospitalização, institucionalização precoce, quedas, perda funcional e óbito^(11)^. Fried et al.^(11)^ propuseram, para identificar a fragilidade, cinco critérios que indicam comprometimento energético como fraqueza muscular, fadiga ou exaustão, lentidão na velocidade da marcha, redução na atividade física e perda de peso não intencional.

Estudos recentes sugerem que a perda auditiva está relacionada com fragilidade e suas consequências^(4,12,13)^. Os primeiros resultados, publicados em 2014^(12)^, demonstraram associação entre a perda auditiva autorreferida e fragilidade em mulheres idosas, porém esta associação não foi encontrada em homens. A perda auditiva foi associada ao risco de desenvolvimento da fragilidade e quedas ao longo dos anos^(13)^, sendo que o risco de progressão da fragilidade foi confirmado nos estudos posteriores^(4,14)^.

Apesar da temática ser de extremo interesse na área do envelhecimento, ressalta-se que os estudos que mostram a relação entre a perda auditiva e a fragilidade ainda são recentes e escassos na literatura. Os estudos prévios utilizaram, como forma de investigação auditiva, pergunta única a respeito da autopercepção auditiva^(4,12)^ ou resultados de avaliações audiométricas^(13)^, contudo nenhum utilizou o HHIE-s^(15)^, instrumento validado e consolidado na literatura internacional para mensurar a desvantagem ou restrição à participação auditiva e os prejuízos sociais e emocionais decorrentes do déficit auditivo. Portanto, o objetivo deste estudo foi verificar a relação entre o *handicap* auditivo e a presença de fragilidade em idosos da comunidade.

## MÉTODO

### Aspectos éticos

O estudo utilizou dados oriundos do projeto “Ferramenta de monitoramento de níveis de fragilidade em idosos atendidos na atenção básica de saúde: avaliação de sua efetividade e eficiência” realizado pelo grupo de pesquisa Gestão em Envelhecimento-GEnv. O projeto foi autorizado pela Secretaria Municipal de Saúde do município e aprovado pelo Comitê de Ética em Pesquisa da Universidade Federal de São Carlos (CAAE:861067418.4.0000.5504 e Parecer nº 3.101.282), respeitando os aspectos éticos previstos pela Resolução 466/2012 regulamentada pelo Conselho Nacional de Saúde.

### Desenho, local de estudo e período

Trata-se de um estudo transversal, observacional, de abordagem quantitativa, realizado em uma extensa comunidade de alta vulnerabilidade social “Cidade Aracy”, localizada no município de São Carlos-SP. A pesquisa foi desenvolvida no período entre 2017 e 2018, sendo avaliados em seus domicílios idosos cadastrados em cinco unidades da Unidade de Saúde da Família (USFs) assistidas pelas administrações regionais de saúde (ARES).

### Amostra, critério de inclusão e exclusão

Os critérios de inclusão foram: possuir idade igual ou superior a 60 anos, ser cadastrado na USFs das ARES “Cidade Aracy” assistidas pelo núcleo de atendimento à saúde da família, ter capacidade de compreensão e comunicação verbal, aceitar participar do estudo e assinar o Termo de Consentimento Livre e Esclarecido. Os critérios de exclusão foram: possuir condições que impedissem a realização dos testes como déficits motores graves, falta de comunicação e doenças em estado terminal. A amostra final foi composta por 238 idosos que preencheram os critérios de elegibilidade para esse estudo.

### Protocolo de estudo

Neste estudo os dados de caracterização sociodemográfica, teste de fragilidade e avaliação do *handicap* auditivo compuseram o protocolo de pesquisa. Os dados sociodemográficos e de saúde foram coletados por meio de questionário construído pelos pesquisadores, com informações sobre sexo, idade, raça, estado civil, escolaridade, renda e comorbidades.

Para os dados da fragilidade foi utilizado o fenótipo proposto por Fried et al.^(11)^, que avalia a fragilidade segundo cinco componentes: 1) Perda de peso não intencional: este critério foi avaliado por meio da seguinte pergunta: “Nos últimos doze meses o (a) senhor (a) acha que perdeu peso sem fazer nenhuma dieta? ” Em caso afirmativo, se essa perda de peso fosse igual ou superior a 4,5 kg ou 5% do peso corporal em um ano, o idoso pontuava neste critério. (2) Fadiga: avaliada por meio do autorrelato evocado por duas questões: “O (a) Sr (a) sentiu que teve que fazer algum esforço para dar conta de suas tarefas do dia-a-dia?” e “O(a) Sr (a) sentiu que não conseguiria levar adiante as suas tarefas?”. Se o idoso respondesse “sempre” ou “na maioria das vezes” para qualquer uma das duas questões, pontuava nesse critério. (3) Baixa força de preensão palmar: foi medida com dinamômetro hidráulico portátil na mão dominante e o idoso foi orientado a apertar o aparelho o mais forte que conseguia. Antes, recebia o comando “atenção, prepara, já! ”, e era estimulado durante o teste com “força, força, força! ”. Para esse critério, o resultado foi obtido pela média aritmética de três medidas, sendo o resultado ajustado segundo o Índice de Massa Corporal e sexo, considerando os 20% mais fracos da distribuição amostral. (4) Baixo nível de dispêndio calórico: este critério foi avaliado por meio de autorrelato a partir da pergunta “O (a) senhor (a) acha que faz menos atividades físicas do que há doze meses atrás? ” Caso a resposta fosse afirmativa, o idoso pontuava neste critério. E (5) lentidão da marcha: avaliada pelo tempo médio gasto para percorrer 4,6 metros de distância, com ajuste segundo sexo e altura. Foram realizadas três medidas da velocidade da marcha, utilizando-se como resposta a média aritmética, considerando os 20% mais lentos da distribuição amostral. De acordo com a pontuação obtida nos cinco critérios, o idoso foi classificado segundo a Fragilidade^(11)^ sendo: Não Frágil (ausência de pontuação nos cinco critérios); Pré-frágil (pontuação em um ou dois critérios); e frágil (pontuação em três ou mais critérios).

Para avaliar o *handicap* auditivo aplicou-se o questionário *Hearing Handicap Inventory for the Elderly - Screening version* (HHIE-S)^(15)^, instrumento traduzido e validado para o Brasil em 1997^(16)^. Este é um instrumento de triagem auditiva para idosos que avalia a percepção do *handicap* auditivo (desvantagem ou restrição à participação auditiva), e o impacto social e emocional decorrente da perda auditiva percebida. Composto por dez perguntas e dividido entre escala social e escala emocional, as respostas de cada item eram sim (4 pontos), algumas vezes (2 pontos) ou não (0 pontos). As pontuações totais variam de 0 a 40 pontos, sendo classificada em três categorias: 0-8 pontos ausência de percepção do *handicap* auditivo, de 10-22 pontos percepção leve ou moderada e de 24-40 pontos percepção significativa do *handicap* auditivo.

### Análises dos resultados e estatística

Antes da digitação dos dados do estudo principal, os protocolos foram conferidos. Os dados foram digitados em duas planilhas diferentes por pesquisadores diferentes e de forma cega no programa MS Excel XP e posteriormente importados e analisados com apoio do pacote estatístico IBM SPSS *Statistics* 20.

Para descrever o perfil da amostra, foi realizada estatística descritiva, com medidas de posição e dispersão (média, desvio-padrão, valores mínimo e máximo, mediana) para as variáveis contínuas. Tabelas de frequências absoluta e relativa foram confeccionadas para as variáveis categóricas. A normalidade dos dados foi avaliada por meio do teste Komogorov Smirnov, seguido dos testes não paramétricos de Kruskal-Wallis, para avaliar a relação entre o *handicap* auditivo e a fragilidade, utilizando os valores médios do HHIE-S para comparar os grupos segundo a fragilidade e Correlação de Sperman, para avaliar a correlação entre o *handicap* auditivo e a fragilidade (variáveis contínuas) e adotou-se a seguinte escala para interpretação dos valores estatisticamente significantes: fraca <0,29; moderada de 0,3 a 0,59; forte de 0,6 a 0,9 e perfeita 1,0^(17)^. Em todas as análises adotou-se p≤ 0,05 para atribuir significância estatística.

## RESULTADOS

A amostra foi constituída por 238 idosos na faixa etária de 60 a 100 anos, com média de 71,9 anos, sendo que 85,7% possuíam idade inferior a 80 anos. Tratou-se de uma população de baixa renda e baixa escolaridade, residentes em uma comunidade considerada de alta vulnerabilidade social no município. A condição de fragilidade e pré-fragilidade esteve presente na maioria da amostra, enquanto 26,9% da população estudada relatou percepção de *handicap* auditivo (Tabela 1).

**Tabela 1:** Caracterização sociodemográfica e de saúde. São Carlos, São Paulo, Brasil, 2018 (n=238)

Variável	Categoria	n	%	Média	DP	Mediana	[Mín-Máx]
Sexo	Feminino	99	41,6				
	Masculino	139	58,4				
Idade (anos)				71,10	7,3	71,0	[60-100]
Faixa etária	60-610 anos	104	43,7				
	71-710 anos	100	42,0				
	80+ anos	34	14,3				
Raça/etnia	Branco	117	410,2				
	Não Branco	121	50,8				
Anos de estudo				2,6	2,8	2,0	[0-17]
N° de pessoas que residem na residência	1-3 pessoas	191	80,3				
	4-5 pessoas	40	16,8				
	6-8 pessoas	7	2,10				
Renda Familiar	R$800 a R$ 1200	41	17,2				
	R$1201 a R$ 2100	66	27,7				
	R$2101 ou mais	67	28,2				
	Não responderam	64	26,1				
			
Comorbidades	Hipertensão arterial	169	71,0				
	Diabetes *Melittus*	166	29,4				
	Osteoporose	38	16,0				
	Depressão	31	13,1				
	Acidente vascular cerebral	23	10,5				
	Cancêr	12	5,5				
			
Handicap Auditivo (HHIE-S)*	Não há prejuízo	174	73,1				
	Prejuízo leve/ moderado	49	20,6				
	Prejuízo significante	15	6,3				
			
Fragilidade	Não frágil	55	23,1				
	Pré frágil	134	56,3				
	Frágil	49	20,6				

* *Hearing Handicap Inventory for the Elderly - Screening version* ^(15)^

A Tabela 2 apresenta a pontuação média no teste HHIE-S em cada grupo composto segundo o critério de fragilidade. Foram encontradas diferenças significativas quanto à pontuação média do HHIE entre idosos não frágeis e frágeis e entre os não frágeis e pré-frágeis.

**Tabela 2:** Comparação entre níveis de handicap auditivo e de fragilidade. São Carlos, São Paulo, Brasil, 2018 (n=238).

	Não-Frágil	Pré-Frágil	Frágil
N	55	134	49
Média HHIE-S*	3,7	6,8	8,9
DP	7,8	9,5	10,8
p-valor NF/PF^a^	0,044**		
p-valor NF/F^a^	0,015**		
p- valor PF/F^a^	0,995		

a Teste Kruskal Wallis

* *Hearing Handicap Inventory for the Elderly - Screening version* ^(15)^;

** Estatisticamente significante

De posse deste resultado, foi utilizado o teste de correlação de Spearman para avaliar se havia correlação estatisticamente significante entre o *handicap* auditivo e a fragilidade, o resultado demonstrou uma correlação positiva entre estas variáveis, tanto para a escala social quanto emocional uma vez que o aumento na pontuação do teste HHIE-S foi acompanhado do aumento na pontuação da fragilidade (r=0,218; p=0,001) (Tabela 3).

**Tabela 3:** Correlação entre prejuízo auditivo e fragilidade. São Carlos, São Paulo, Brasil, 2018 (n=238)

Prejuízo auditivo (HHIE-S)*	Fragilidade	
	*r*	*p*
	0,218	0,001**
Escala Social	0,228	0,001**
Escala Emocional	0,200	0,002**

Correlação de Spearman

* *Hearing Handicap Inventory for the Elderly - Screening version* ^(15)^;

** Estatisticamente significante

## DISCUSSÃO

Este estudo se propôs investigar a relação entre o *handicap* auditivo e a fragilidade em 238 idosos da comunidade. Os resultados demonstraram que idosos frágeis e pré-frágeis relatam restrição de participação em atividades de vida diária segundo o questionário HHIE-S. Tal resultado foi ratificado pela correlação positiva entre a pontuação da fragilidade, segundo o Fenótipo de Fried, e a pontuação do HHIE-S.

O instrumento HHIE-S utilizado neste estudo tem o objetivo de quantificar o impacto social e emocional advindo da perda auditiva, sendo um instrumento útil para rastreio auditivo e mensuração de respostas a tratamentos e *follow-up*, tanto na clínica quanto em pesquisas^(18)^. Assim como a presença de queixa auditiva autorrelatada, a pesquisa do *handicap* auditivo é uma forma de avaliação subjetiva da audição comumente utilizada nos estudos populacionais nacionais^(5)^ e internacionais^(4)^ que realizam coletas domiciliares que visam investigar as restrições decorrentes de problemas auditivos. Tratam-se de questões previamente validadas em estudos anteriores que tiveram sua acurácia testada quando comparada a outras formas de avaliação objetiva^(16,18)^.

Com a publicação da CIF em 2001 e a ampliação de seu uso ao longo destas duas décadas, a perda auditiva passou a ter uma abordagem biopsicossocial focada na promoção de saúde e bem-estar, com a incapacidade construída dentro da interação pessoa-ambiente. Ferramentas que se propunham a avaliar a autopercepção auditiva em atividades em vida diária, ganharam maior relevância uma vez que possibilitam a avaliação da restrição à participação auditiva^(9,19)^. A audiometria tonal liminar dentre outras medidas da sensibilidade auditiva, apesar de sua importância ímpar, não são suficientes para mensurar a desvantagem auditiva, ou seja, como o indivíduo se adapta a sua deficiência, ou qual o impacto na qualidade de vida. Assim, a opção de se estudar o *handicap* auditivo e sua relação com a fragilidade em idosos transcende a mera constatação da perda auditiva e considera o indivíduo como um ser biopsicossocial, inserido num contexto ambiental próprio, com habilidades para se adaptar à sua deficiência/incapacidade, o que justifica a relação encontrada entre a restrição da participação auditiva e a fragilidade em idosos.

A fragilidade foi investigada por meio do instrumento proposto por Fried et al.^(11)^ que apontou elevado número de idosos pré-frágeis e frágeis, totalizando 76,10% da amostra, prevalência similar a outros estudos da literatura nacional e internacional que utilizaram o mesmo instrumento de avaliação da Fragilidade na comunidade^(13,20)^.

Ao investigar a relação entre o HHIE-S e a fragilidade foi possível verificar que indivíduos pré-frágeis e frágeis possuem maior restrição de participação em atividades de vida diária comparados aos não frágeis (p=0,044 e 0,015, respectivamente). No entanto, os grupos pré-frágil e frágil não diferiram entre si, quando se comparou a média do HHIE-S. É importante destacar que em estudos longitudinais foi observado que a perda auditiva aumenta o risco de incidência e progressão da fragilidade^(4,12,14)^.

Da mesma forma, os resultados demonstram que a pontuação do HHIE-S apresentou correlação positiva estatisticamente significante com a pontuação da fragilidade (r=0,218 e p=0,001), sendo essa correlação demonstrada tanto na escala social quanto na emocional do *HHIE-S*. Embora as correlações tenham sido de fraca magnitude, tais achados sugerem que quanto maior a restrição de participação em atividades de vida diária maior a pontuação indicativa de fragilidade, sendo a força dessa correlação semelhante nas escalas social e emocional.

O estudo aqui apresentado se mostra bastante atual pois publicações sobre o tema perda auditiva e fragilidade são escassas na literatura e os resultados apresentados são condizentes com os achados anteriores^(4,12,13,21)^, demonstrando a pertinência da pergunta de pesquisa. Não foram encontrados estudos nacionais ou realizados na língua Portuguesa que abordassem o tema. O estudo mais remoto identificado na literatura que tratou diretamente do tema fragilidade e perda auditiva foi publicado em 2014^(12)^ seguido de uma publicação em 2015^(13)^ outra em 2017^(4)^, sendo que, nestes estudos, apesar de utilizarem metodologias diferentes, houve associação entre perda auditiva e fragilidade. No entanto, os autores destacaram as condições de saúde adversas como diabetes, doenças cardiovasculares ou história de quedas como fatores associados à fragilidade^(13)^ e/ou perdas auditivas^(4,22)^. As explicações potenciais para esta associação incluem uma etiologia neuropatológica compartilhada (por exemplo, doença microvascular e inflamação) contribuindo tanto para a deficiência auditiva, quanto para a fragilidade^(12)^.

A deficiência auditiva também já foi associada com maior risco de mortalidade por doenças cardiovasculares, sendo que quando associada a outras deficiências sensoriais, como a visual, aumentou-se o risco de mortalidade por todas as causas, possivelmente porque é comum pessoas mais velhas terem deficiências sensoriais múltiplas, podendo haver condições de saúde adversas o que aumenta o risco de fragilidade e consequente morte^(12,22,23)^.

A associação entre perda auditiva e mortalidade também foi estudada^(23)^, concluindo que a perda auditiva em idosos pode estar associada ao aumento de até 20% no risco de mortalidade, podendo ser explicado pelos processos patológicos compartilhados como disfunção mitocondrial, inflamação generalizada ou doença microvascular podendo contribuir potencialmente para uma perda auditiva e risco de mortalidade.

Outras variáveis sociodemográficas como idade, sexo, renda, educação e suporte social também foram consideradas nestes estudos prévios de perda auditiva e fragilidade. Em um estudo transversal realizado com 2.1010 idosos acima de 70 anos, quando feita a estratificação por sexo, a audição foi significativamente associada à fragilidade em mulheres, porém não em homens^(12)^. Um estudo de coorte sobre o tema foi publicado em 2017^(4)^, utilizando uma pergunta de autoavaliação para identificação da perda auditiva e o fenótipo de Fried, para identificação da fragilidade, o que caracterizou o estudo como inédito. Foram acompanhados 2.836 idosos que tiveram a audição autoavaliada por meio de uma única questão em que os idosos deviam classificá-la como excelente, muito boa, boa, razoável ou ruim. As respostas foram organizadas compondo dois grupos de análise: audição boa (resposta excelente, muito boa, boa) e audição ruim (resposta razoável e ruim). A pesquisa analisou os dados basais de forma transversal e reavaliou os idosos após quatro anos. Para estabelecer a associação entre perda auditiva e fragilidade, o estudo levou em consideração ajuste de sexo, idade, renda, educação, doenças cardiovasculares, cognição, depressão e socialização e revelou que a perda auditiva foi associada com fragilidade e pré-fragilidade no estudo de base (corte transversal). Tal associação foi confirmada na análise longitudinal no qual a perda auditiva em idosos pré-frágeis foi associada ao maior risco de progressão da fragilidade, independentemente das covariáveis. Para os autores, perda auditiva em idosos pode ser um risco para fragilidade, sugerindo que a deficiência auditiva acelera a progressão da fragilidade^(4)^.

As limitações comunicativas impostas pela perda auditiva provocam isolamento social e familiar, com restrição à participação auditiva e nas atividades de vida diária, como ir ao supermercado, igrejas, reuniões. Tal situação impacta não somente nas atividades sociais, mas também provoca redução dos níveis de atividade física e qualidade de vida^(24)^. Assim, é fundamental considerar as intervenções necessárias, como a adaptação de Aparelho de Amplificação Sonora Individual (AASI), para a reabilitação auditiva, com vistas a melhorar a autopercepção do *handicap* e qualidade de vida do idoso^(25)^.

Por fim, observou-se que as comorbidades mais prevalentes foram hipertensão arterial (71%) e diabetes mellitus (29,4%). As características sociodemográficas como idade, sexo, escolaridade, renda e etnia também foram levantadas neste estudo, porém não foram consideradas para análise de fatores associados a perda auditiva ou fragilidade, sendo essa uma limitação do presente estudo.

Assim, apesar dos estudos prévios demonstrarem que a relação entre perda auditiva e fragilidade se mantém mesmo após os ajustes dos fatores associados, os resultados da presente pesquisa não permitem afirmar que as relações demonstradas entre as duas variáveis não estejam mascaradas por fatores sociodemográficos ou condições de saúde que interferem tanto na audição quanto na fragilidade de idosos. Por se tratar de um estudo transversal, também não se pode indicar a relação de causalidade entre as duas variáveis, como pôde ser demonstrado nos estudos de coorte^(4,12)^.

Apesar disso, aventa-se a possibilidade desta relação ser decorrente dos desfechos negativos da perda auditiva, como a restrição de participação em atividades de vida diária, dificuldade de comunicação e consequente isolamento social, levando ao desencadeamento e progressão da fragilidade^(13)^. Assim, este estudo se mostra importante uma vez que vem ratificar a relação entre perdas auditivas e fragilidade, uma temática tão recente e pouco explorada, que demonstra a importância do monitoramento auditivo em idosos. A restrição da participação em atividades de vida diária, em decorrência da perda auditiva, também pode ser um importante componente de avaliação quando se objetiva identificar indivíduos com risco de fragilidade^(4)^.

A partir desses achados, vários caminhos da pesquisa podem ser explorados. Sugere-se analisar como os cinco componentes incluídos no fenótipo de fragilidade segundo Fried se relacionam individualmente com a audição.

Os resultados analisados nesta pesquisa foram baseados em autoavaliação da audição, mais especificamente na identificação do *handicap* auditivo, ou seja, restrição de participação em atividades de vida diária e seus impactos sociais e emocionais decorrentes da privação auditiva percebida. No entanto, a avaliação auditiva considerada padrão ouro é a Audiometria Tonal Liminar, exame que detecta a perda auditiva e mensura seu grau. Pesquisas anteriores apontam que a prevalência de perdas auditivas autodeclaradas nos estudos populacionais é inferior às perdas auditivas aferidas por meio de avaliações audiométricas^(18)^.

Apesar dos resultados promissores, não conhecemos as características auditivas que de fato se relacionam com a fragilidade, no que diz respeito ao grau e compreensão de fala. Também não sabemos como a reabilitação auditiva, mais especificamente o uso de próteses auditivas, pode interferir nesta relação. Assim, pesquisas futuras poderão explorar a relação da fragilidade com as diferentes configurações auditivas e como a reabilitação auditiva pode impactar no quadro de fragilidade. Mais pesquisas poderiam auxiliar no aprofundamento do tema e direcionamento de estratégias de controle da progressão da fragilidade.

## CONCLUSÃO

Os resultados apresentados neste estudo demostram que há relação entre a percepção subjetiva do *handicap* auditivo e a fragilidade em idosos da comunidade.

O presente estudo se faz relevante perante sua inovação em correlacionar a perda auditiva, mais especificamente a restrição da participação em atividades diárias, com a fragilidade de idosos cadastrados na atenção básica e em contexto vulnerável. Outra potencialidade do estudo é a sua contribuição para o serviço de saúde com a utilização dos achados para a elaboração de estratégias de monitoramento de saúde da pessoa idosa com vistas à garantia do cuidado integral.
